# Assessment of anticancer properties of cumin seed (*Cuminum cyminum*) against bone cancer

**DOI:** 10.3389/fonc.2023.1322875

**Published:** 2023-12-06

**Authors:** Rajkuberan Chandrasekaran, Muthukumar Krishnan, Sonu Chacko, Omkar Gawade, Sheik Hasan, John Joseph, Evelin George, Nemat Ali, Abdullah F. AlAsmari, Sandip Patil, Haoli Jiang

**Affiliations:** ^1^ Department of Biotechnology, Karpagam Academy of Higher Education, Coimbatore, India; ^2^ Department of Petrochemical Technology, Anna University, Tiruchirappalli, India; ^3^ Department of Biochemistry, JSS Academy of Higher Education, Mysuru, India; ^4^ Department of Pharmacology and Toxicology, College of Pharmacy, King Saud University, Riyadh, Saudi Arabia; ^5^ Department of Haematology and Oncology, Shenzhen Children’s Hospital, Shenzhen, China; ^6^ Department of Orthopedics, the Third People’s Hospital of Shenzhen, Shenzhen, China

**Keywords:** cumin seed, anticancer, bone, cancer, bacteria, MDR strains

## Abstract

**Introduction:**

Early-life osteosarcoma is associated with severe morbidity and mortality, particularly affecting young children and adults. The present cancer treatment regimen is exceedingly costly, and medications like ifosfamide, doxorubicin, and cisplatin have unneeded negative effects on the body. With the introduction of hyphenated technology to create medications based on plant molecules, the application of ayurvedic medicine as a new dimension (formulation, active ingredients, and nanoparticles) in the modern period is rapidly growing. The primary source of lead compounds for the development of medications for avariety of ailments is plants and their products. Traditionally, *Cuminum cyminum* (cumin) has been used as medication to treat a variety of illnesses and conditions.

**Methods:**

The cumin seed was successfully extracted with solvents Hexane, Chloroform, Methanol, Ethanol and Acetone. Following the solvent extraction, the extract residue was assayed in MG63 cells for their anti-proliferative properties.

**Results:**

First, we used the [3-(4,5-Dimethylthiazol-2-yl)-2,5-Diphenyltetrazolium Bromide] (MTT) assay to test the extracted residue’s cytotoxicity. The results show that hexane extract Half-maximal inhibitory concentration (IC50 86 µG/mL) effciently inhibits cells by causing programmed cell death. Furthermore, using the Acridine orange/ethidium bromide (AO/EB) staining method, the lactate dehydrogenase assay, and the reactive oxygen species assay using the Dichloro-dihydro-fluorescein diacetate (DCHFDA) staining method, we have demonstrated that the hexane extract causes apoptosis in MG63 cells. Furthermore, flow cytometry research revealed that the hexane extract stops the cell cycle in the S phase. In addition, the hexane extract limits colony formation and the migration potential as shown by the scratch wound healing assay. Furthermore, the extract from cumin seeds exhibits remarkable bactericidal properties against infections that are resistant to drugs. Gas chromatography analysis was used to quantitatively determine the hexane and methanolic extract based on the experimental data. The primary chemical components of the extract are revealed by the study, and these help the malignant cells heal. The present study finds that there is scientific validity in using cumin seeds as a novel method of anticancer therapy after undergoing both intrinsic and extrinsic research.

## 1 Introduction

Osteosarcoma is a bone cancer arising at the ages of 10–14, specifically in children and adolescents, and, even at the age of 50, there is a high risk of suffering osteosarcoma (1). The primary change in the metaphysis of the long bones, followed by accelerated cell division, is responsible for osteosarcoma (2). At this stage, the loss of functionality of the tumor suppressor gene will eventually develop into cancer. In India, the incidence of these cancers varied from 4.7% to 11.6%, with significant morbidity and mortality. However, in recent 5-year reports, the occurrence of these cancers rose to 44% in India compared with that in other countries (3). Henceforth, the demographic data suggest that it is indeed to initiate modernized chemotherapies to counteract the diseases intensively.

Osteosarcoma is now treated with chemotherapy and surgery, as well as medication such as methotrexate, doxorubicin, ifosfamide, and cisplatin (4). However, 70% of patients now have a survival of up to 5 years because of this medication. In addition, acquired chemoresistance has been linked in certain instances to poor prognosis, with a survival rate of only 20 years (5). Creating medications with significant long-term effects and lower toxicity is indeed required to overcome these obstacles and other related side effects.

Many purgative qualities are available for a range of illnesses and disorders through plants and their products. In the past, Ayurveda, an indigenous medical system, was widely utilized throughout the nation (6). All infectious and non-infectious disorders were treated with great efficacy and zero toxicity by Ayurvedic medicine (7). Undeniably, plants are versatile resources that can be developed as lead molecules for treating cancers. For instance, camptothecins (irinotecan and topotecan), taxanes (paclitaxel), epipodophyllotoxins (etoposide), and *Vinca alkaloids* (vincristine and vinblastine) were the drugs derived from the plants that are widely in various types of cancer chemotherapy (8). Plants contain phenols, alkaloids, terpenoids, tannins, proteins, and other biomolecules involving antiproliferative properties (9). Henceforth, understanding the plant’s chemical entities and exploring its prospective applications in cancer therapy will eventually develop new drugs with high efficacy and efficiency.

Indian spices are fragrant and have a wide range of purgative qualities that can be used to treat anything from tumors to the common cold (10). Indians use spices as a food element in a variety of culinary preparations. Curcumin, derived from *Curcuma longa*, is a traditional example of Indian spice and an essential anticancer agent against a variety of cancers (11). Similarly, antiproliferative qualities were discovered for capsicum, ginger, garlic, fenugreek, bay leaves, cinnamon, and cumin (12).

Cumin seeds (*Cuminumcyminum* L.) belong to the family of *Apiaceae*; the seeds are an aromatic spice that imparts taste, color, and flavor to food preparations (13). Apart from food preparations, the seed has distinctive medicinal properties due to the rich content of phenols, flavanoids, and terpenes (14). The cumin exhibited strong antagonistic activity against bacterial and fungal pathogens, viral, anticarcinogenic, wound healing, antioxidant, ovicidal, and hypoglycemic activities (15). At the present epoch, microbes tend to acquire resistance against antibiotics, which is a serious concern to the research fraternities (16). The drug-resistant microbes cause severe morbidity in patient’s compliance with existing chronic diseases. Therefore, the development of plant-based chemical moieties as a drug will be sustainable for the management of infectious diseases (17). The choice of cumin seed against bone cancer is the incidence of bone cancer in Indians, whereas the cumin seed is used in the food preparations. Henceforth, the clinical relevance of cumin seeds must be critically investigated. With the above rationale, the current studies manifest the anticancer activities of cumin seeds in the MG63 cell line. Although there are consistent reports of cumin seed extracts in various cancers, our study is the first instance to outcome the anticancer activity of cumin seed extract in the osteoblastic model MG63 cell line.

## 2 Materials and methods

Cell line, chemicals, and reagents were purchased from the authorized agency National Centre for Cell Science (NCCS) Pune India and Sigma-Aldrich Pvt Ltd. Cumin seeds were purchased from local markets in Coimbatore, India.

### 2.1 Preparation of cumin seed extract

Fresh cumin seeds, free from infection, were purchased and transferred immediately to the laboratory. The seeds were rinsed in deionized water and incubated at 50°C for 24 h and macerated in a blender to obtain fine powder formation. A simple infusion technique is used in the present study to prepare the extracts. Solvents such as ethanol (E), methanol (M), acetone (A), ethyl acetate (EA), and n-hexane (NH) were used in the present study. The rationale for using the solvents was based on the polarity: polar solvents (alcohol), intermediate solvent (A); non-polar solvents (chloroform, hexane). For extract preparation, 100 gm of cumin seed is mixed with 250 mL of respective solvents individually. After the extract preparation, using the rotary vacuum evaporator, the extracts were concentrated to form crude extracts. The obtained extracts were stored in a refrigerator and proceeded for further activities.

### 2.2 Antibacterial activity of the crude extract

Multidrug-resistant (MDR) strains—*Bacillus flexus* (*B. flexus*) (NCBI accession number MN045189), *Bacillus filamentosus* (*B. filamentosus*) (MN045186), *Pseudomonas stutzeri* (*P. stutzeri*) (MN045185), and *Acinetobacter baumannii* (*A. baumannii*) (MN045188)—were gifts from the Department of Marine Sciences, Bharathidasan University, Tiruchirappalli, India. The disc diffusion method evaluated the cumin seed extracts against the MDR strains (18). Briefly, a sterile nutrient agar plate was inoculated with 1.5 × 10^8^ colony-forming units (CFU/mL) MDR strains, and different concentrations of cumin seed extracts (25 μg mL^−1^, 50 μg mL^−1^, 75 μg mL^−1^, and 100 μg mL^−1^) were loaded onto the well and incubated at room temperature at 37°C. After the incubation, the zone of inhibition (ZOI) was measured.

### 2.3 Anticancer activity of cumin seed extract

#### 2.3.1 MTT assay

After the collection of MG63 cells from the NCCS, the cells were maintained in Dulbecco’s modified Eagle’s medium (DMEM) and kept in a humidified 5% CO_2_ incubator at 37°C. After 2 days, the monolayer culture cells were trypsinized and suspended in a 10% growth medium; 100 µL of cell suspension was added to a multi-well plate and incubated in a CO_2_ incubator.

By using cyclomixer, 1 mg of cumin seed extract was suspended in Dimethyl sulfoxide (DMSO). A 0.22-µm Millipore syringe filter was used to filter the cumin seed extract to ensure sterility. In the 96-well plates, to the cells, different concentrations of the cumin seed extract (6.25 µg/mL, 12.5 µg/mL, 25 µg/mL, 50 µg/mL, and 100 µg/mL) were added and kept in the incubation. After the incubation, 30 µL of MTT solution was added to all the wells and incubated in a CO_2_ incubator for 5 h at 38°C. After the time, to the multi-well plate, 100 µL of Dimethyl sulfoxide (DMSO) was added to solubilize the formazan crystals. At 540-nm wavelength, the absorbance values were measured by using a spectrophotometer (19).

On the basis of the results of the crude extracts, the best solvent extract with a significant Lethal Concentration 50 (LC_50_) value was chosen and proceeded further for other activities.

#### 2.3.2 AO/EB staining

The cell line cultured in an animal tissue culture flask supplemented with DMEM was maintained in a CO_2_ incubator. After attaining 80% confluence, cells were exposed to Lethal Dose 50% (LD_50_) concentration of cumin extract (NH, 86.13 µg/mL) and incubated for 24 h. The cells were bathed with cold Phosphate-buffered saline (PBS) and stained with Ethidium Bromide (EtBr) (100 μg/mL) and AO (100 μg/mL) at 37°C for 20 min (20). Furthermore, the stained cells were washed and subjected to fluorescence microscopic study by using an Olympus fluorescence microscope.

#### 2.3.3 Lactate dehydrogenase assay

The cultured cells supplemented with DMEM were maintained in a CO_2_ incubator. The test was performed with supernatant collected from tissue culture plates which were exposed to different concentrations of hexane extract (6.25 µg/mL, 12.5 µg/mL, 25 µg/mL, 50 µg/mL, and 100 µg/mL). For Optical Density (OD) analysis, a 50-µL sample was added to 1 mL of working reagent and recorded at 340 nm in a spectrophotometer after 1 min of incubation (21).

The activity of lactate dehydrogenase was calculated by using the following formula:


lactate dehydrogenase assay(LDH)activity(U/mL)=((ΔOD)/min.X3333).

#### 2.3.4 *In vitro* ROS measurement using DCFDA staining

The cultured cells supplemented with DMEM were maintained in a CO_2_ incubator. The cells were washed and stained with DCFDA, and a 50-µL sample was added and incubated for 35 min (22). After incubation, the unbound dye was washed with PBS, and the fluorescence was captured by Olympus fluorescence. At 470-nm excitation and 635-nm emission, the intensity of fluorescence was measured and expressed in arbitrary units (AU).

### 2.4 Cell cycle analysis

The MG63 cells at 1 × 10^6^ were cultured in six-well plates and incubated with NH extract of 86.13 µg/mL for 24 h. After the period, the cells were washed, centrifuged, resuspended in E, and kept incubated at −20°C. After incubation, the cells were centrifuged, and, to the pellet, PBS and cell cycle reagent of 250 µL were added and incubated in the dark for 30 min (23). A flow cytometer was used to analyze the treated and untreated cells.

### 2.5 Scratch assay

For the assay, MG63 cells at a density of 300,000 cells per well were seeded into a multi-well plate for 24 h of incubation (24). Using a sterile pipette tip, a wound was made by scratching the cells. After scratches, the debris was removed and the cell was washed with PBS, followed by incubation with NH extract of 86.13 µg/mL at varying time intervals (0 h–24 h–48 h–72 h–96 h). The wound areas were annotated by capturing the images, and the effect of the hexane extract on wound closure was determined microscopically (×4 magnification, Olympus CKX41) and calculated using MRI-ImageJ analysis software.

### 2.6 Clonogenic assay

The cells at a density of 1 × 10^3^ cells were treated with the hexane extract and kept in a CO_2_ incubator for 24 h. After that time, the medium was replenished with the new medium and again incubated for 5 days. After the period, the medium was changed and washed with PBS buffer, and the cells were fixed with fixative formaldehyde for 2 h at 37°C and stained with crystal violet for 30 min. The assay was made in triplicate, and colonies with 50 cells were counted (25).

### 2.7 GC-MS analysis

The Gas chromatography-Mass spectrometry (GC-MS) analysis was done for the extracts of M and NH. The analysis was performed with the instrument GC-MS QP 2010 (Shimadzu) with the standard operating conditions and analyzed with the previously reported methodology (26). The results were compared, and the compounds were identified using the Spectral Library search program of the National Institute of Standards and Technology (NIST).

## 3 Results and discussion

Humans underuse, overuse, and misuse antibiotics, which pave the way for developing resistance in microbes (27). In general, MDR strains cannot be controlled with standard drugs, increasing the risk of infection to others (28). The globally emerging MDR strains make the treatment difficult to control and speed up the infection persistency (29). Henceforth, the emergence of these superbugs should be controlled with the development of novel antimicrobial drugs from natural bioresources. Plants with diverse, unique natural secondary metabolites possess various therapeutic properties. MDR strains can be substantially controlled by phytochemicals derived from plant resources effectively.

### 3.1 Antimicrobial activity

**Figure 1:**
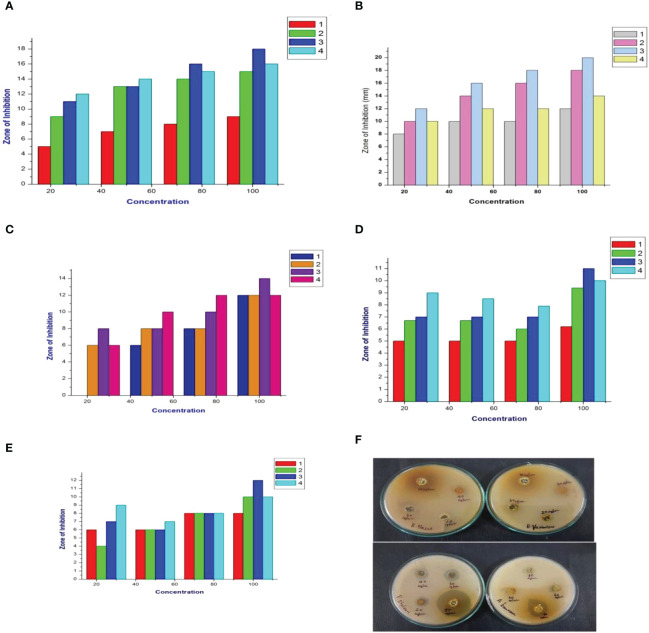
Antimicrobial activity of cumin seed extracts against MDR strains. **(A)** Graphical representation of the hexane extract (ZOI) in mm. **(B)** Graphical representation of the methanol extract (ZOI) in mm. **(C)** Graphical representation of the ethyl acetate extract (ZOI) in mm. **(D)** Graphical representation of the ethanol extract (ZOI) in mm. **(E)** Graphical representation of the acetone extract (ZOI) in mm. (1) *Bacillus flexus* (MN045189), (2) *Bacillus filamentosus* (MN045186), (3) *Pseudomonas stutzeri* (MN045185), and (4) *Acinetobacter baumannii* (MN045188). **(F)** Photograph of the methanol extract against MDR strains.

The present study evaluated the cumin seed extracts against the MDR pathogen B. *flexus* (MN045189), *B. filamentosus* (MN045186), *P. stutzeri* (MN045185), and *A. baumannii* (MN045188) by the disc diffusion method. As a result, the cumin extracts displayed strong antagonistic activity against the MDR pathogens. As illustrated in **Figure 1**, at varying dosage levels (25 µg/mL to 100 µg/mL), the cumin extracts performed concentration-dependent activity against the bacterium. The order of efficacy was observed to be high in M extract > ethanol extract > hexane extract > A extract > EA. The M extract performed superior activity against all the tested pathogens. In particular, the M extract displayed stupendous activity in *P. stutzeri > B. filamentosus > A. baumannii > B. flexus.* In our observation, the M extract performed effectively inhibitory against the tested pathogens. This is due to the presence of biomolecules trapped in the M solvent. The reason for the elevated bactericidal activity is M is a polar solvent that can efficiently trap the low–molecular weight polyphenols from the cumin seed. Therefore, the active constituents in the M extract interact with various biomolecules (DNA/RNA/proteins) of bacterial cells and inhibit the functions, which lead to cell death (30) A similar kind of pattern of observations was noticed in the bactericidal activity of Cuminumcyminum oil against MDR *S. aureus* (31). Likewise, consistent reports documented *C. cyminum* oils as strong bactericidal agents in variant pathogens (32). Still, our study is the first to demonstrate cumin seed extract’s antibacterial activity against MDR pathogens.

### 3.2 Anticancer activity of cumin seed extract

#### 3.2.1 Cytotoxicity assay

**Figure 2:**
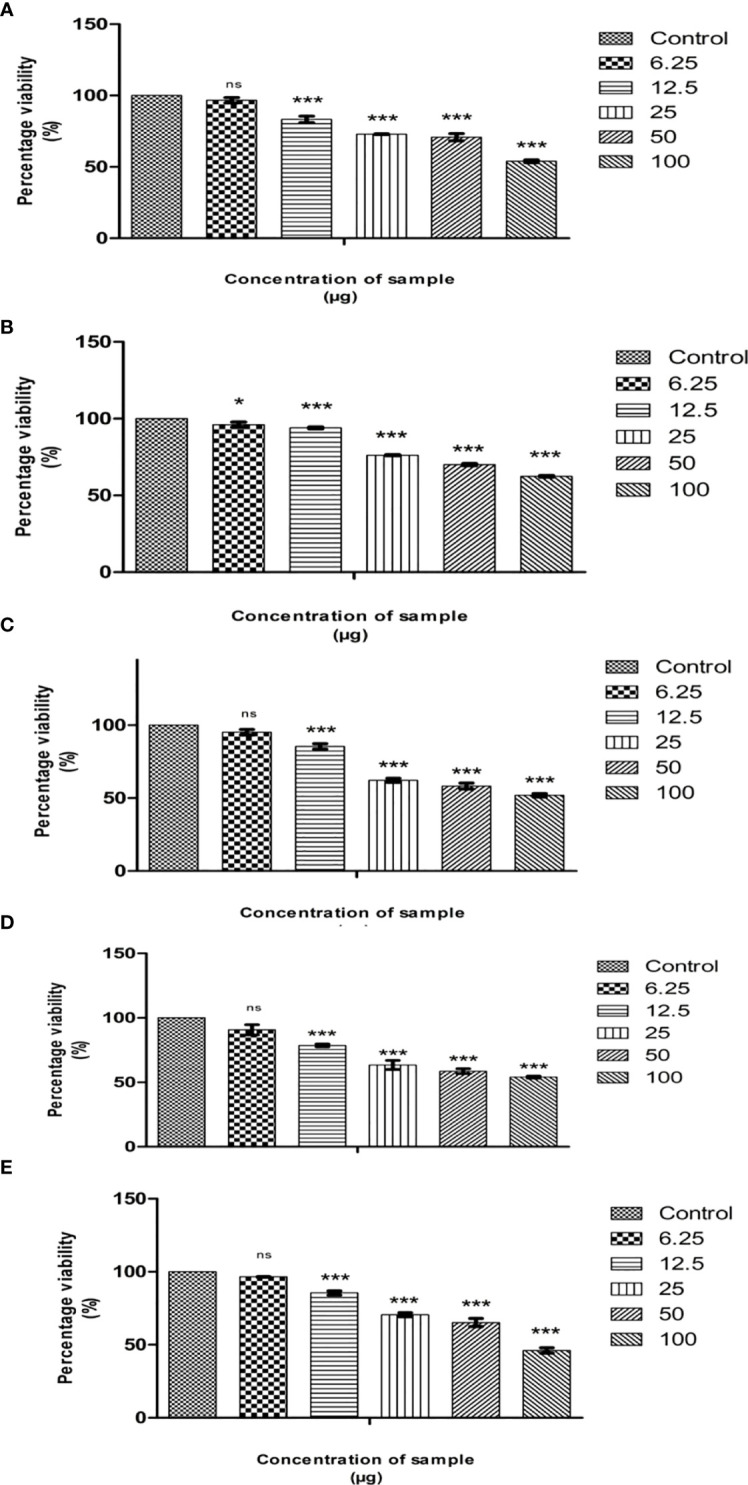
Cytotoxic effects of Cumin seed extract in MG63 cells by MTT assay. **(A)** Hexane extract; **(B)** Methanol extract; **(C)** Ethyl acetate extract; **(D)** Ethanol extract; **(E)** acetone extract Different concentrations (6.25µg, 12,5µg, 25µg, 50 µg and 100 µg) of n hexane extracts were assessed in MG63 cells. The X axis is percentage viability and Y represents the concentration of the sample. All experiments were done in triplicates and results represented as Mean+/- SE. One-way ANOVA and Dunnets test were performed to analyse data. *p< 0.05, ***p< 0.001 compared to control group, ns – non significant

The cytotoxicity of cumin seed (E, M, EA, NH, and A) extracts was evaluated by MTT assay against the MG63 cell line. The MTT assay is a preliminary assay to intrigue the toxic potential of the extracted solvent residue against the cell line. The cumin seed (E, M, EA, NH, and A) extracts at the respective concentrations (6.25 µg/mL to 100 µg/mL) were evaluated, and the result is displayed in **Figures 2A–E**. The cumin seed extract performed significant inhibitory activity against the MG63 cells to dose concentration. **Table 1** determines the cytotoxicity analysis of the crude extracts. Among the extracts, NH extract accelerated the inhibitory activity effectively with a noticeable LC_50_ value. This is due to the synergistic interaction of phytoconstituents in the NH extract, and the NH solvent traps the oil constituents very effectively from the cumin seed. Due to the presence of volatile and non volatile oils and lipophillic compounds the anticancer activity was escalated in the present study. The NH extract interacts with the cell membrane and induces reactive oxygen species (ROS), which substantially interacts with DNA/RNA/proteins and induces apoptosis (33).

**Table 1:** Cytotoxicity analysis of cumin seed extracts against MG63 cells.

S. no	Name of the extract	LC_50_ value
**1. **	**Ethanol**	**113 µg/mL**
**2. **	**Acetone**	**118 µg/mL**
**3. **	**Ethyl acetate**	**130 µg/mL**
**4. **	**Methanol**	**166 µg/mL**
**5. **	**n-Hexane**	**86 µg/mL**

The anticancer activity of solvent extract residue is directly related to the nature of phytochemicals, cell lines, and other factors. Prakash et al. (34) studied the anticancer activity of cumin seeds against seven cell lines, including OVCAR-5, PC-5, SF-295, and Colon 502713, to justify the cell lines Colo-205, Hep-2, and A-549. The ethanolic extract was assayed against the cell line at 100 µg/mL. The ethanolic extract performed effective growth inhibitory activity in different cell lines with significant percentage growth inhibition; the percentage inhibition was high in Colon 502713 cell line at 61% and least in the SF-295 cell line at 25%. Likewise, the benzene extract of cumin seed was tested for cytotoxicity against the six cell lines: HEPG2, HELA, HCT116, MCF7, HEP2, and CACO2 (35). The assay outcome showed a higher growth inhibition in MCF7, followed by HEPG2, HEP2, CACO2, HCT116, and HELA cell lines. The benzene extract does not infer cytotoxic properties against the normal fibroblast cell line BHK. In the present study, we have observed that non-polar NH extract performed superior activity than E, A, EA, and M extracts in the MG63 cell line. Our study is the first to encounter the anticancer activity of cumin seed extract against the MG63 cell line.

#### 3.2.2 AO/EB staining

The most promising effect of herbal therapeutics in cancer cells is to induce apoptosis (36). Chemotherapeutic drugs exert their anticancer activity by activating the apoptosis process. The degree of apoptosis is strongly associated with drug sensitivity; to detect the cell death caused by apoptosis, various techniques like terminal deoxynucleotidyl, propidium iodide, *in situ* nick translation, thermal denaturation assays, and acidic denaturation were employed (37). However, the techniques have limits and delimit, restricting the detection of apoptotic cells. Dual AO/EB staining is a method to determine apoptotic cells (38). The staining method utilizes fluorescent dyes to identify the cell membrane changes associated with apoptosis precisely.

**Figure 3:**
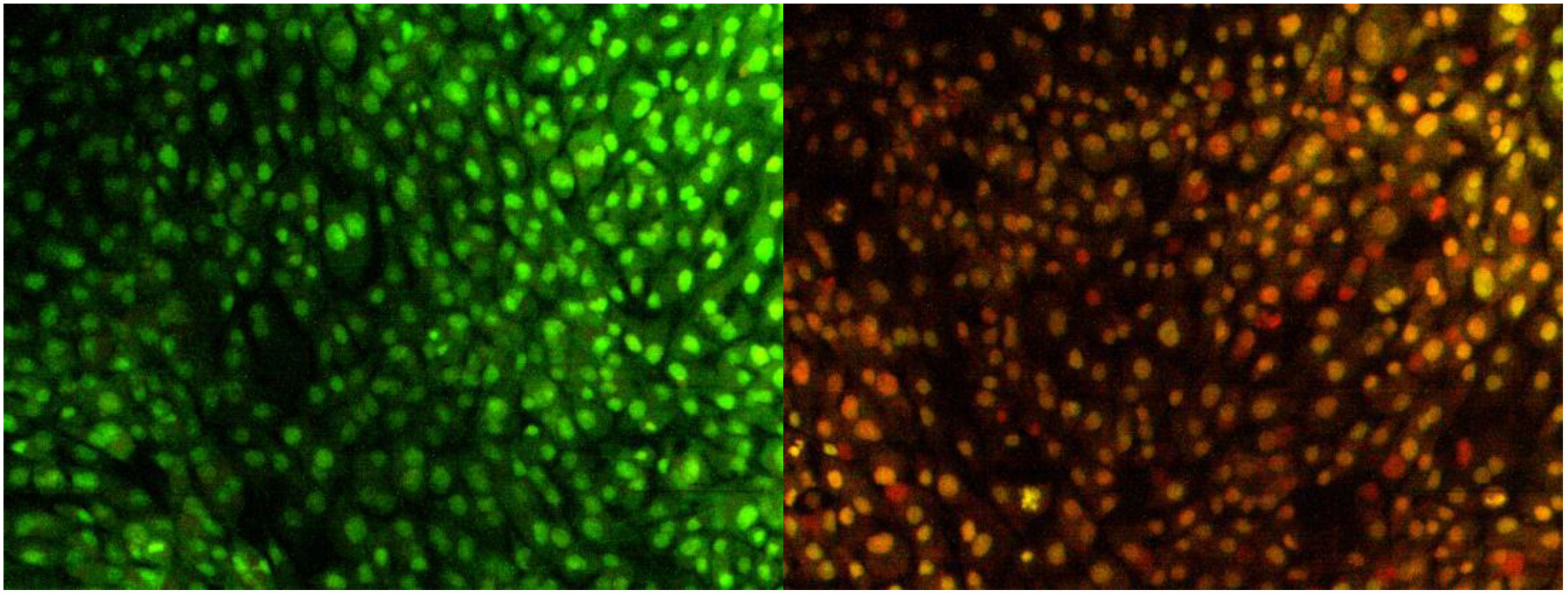
Apoptosis determination by AO/EB staining. Image captured by a fluorescence microscope. The left slide denotes the live cells bright green color (control); right slide denotes the treatment of hexane extract in the cells; green-colored cells, early apoptotic cells; orange-colored cells, late apoptotic cells.

In the present study, the effect of NH extract on the MG63 cell line was subjected to AO/EB staining. On illumination with a fluorescent microscope, the images were captured and presented as **Figure 3**. The left side of the image depicts the control group; green-colored cells indicate that a circular nucleus is uniformly distributed in the center of the cell. The right side of the image denotes the apoptotic, late apoptotic, and necrotic cells. Early apoptotic cells were denoted by orange-green fluorescence, whereas orange fluorescence implies late apoptotic cells; red fluorescence denotes the necrotic cells. AO can easily penetrate the live cells, whereas EB will penetrate only to the dead cells where the cell membrane is ruptured (39). From the image, it is assumed that NH extracts trigger apoptosis in the MG63 cells and cause cell death.

#### 3.2.3 Lactate dehydrogenase assay

**Figure 4:**
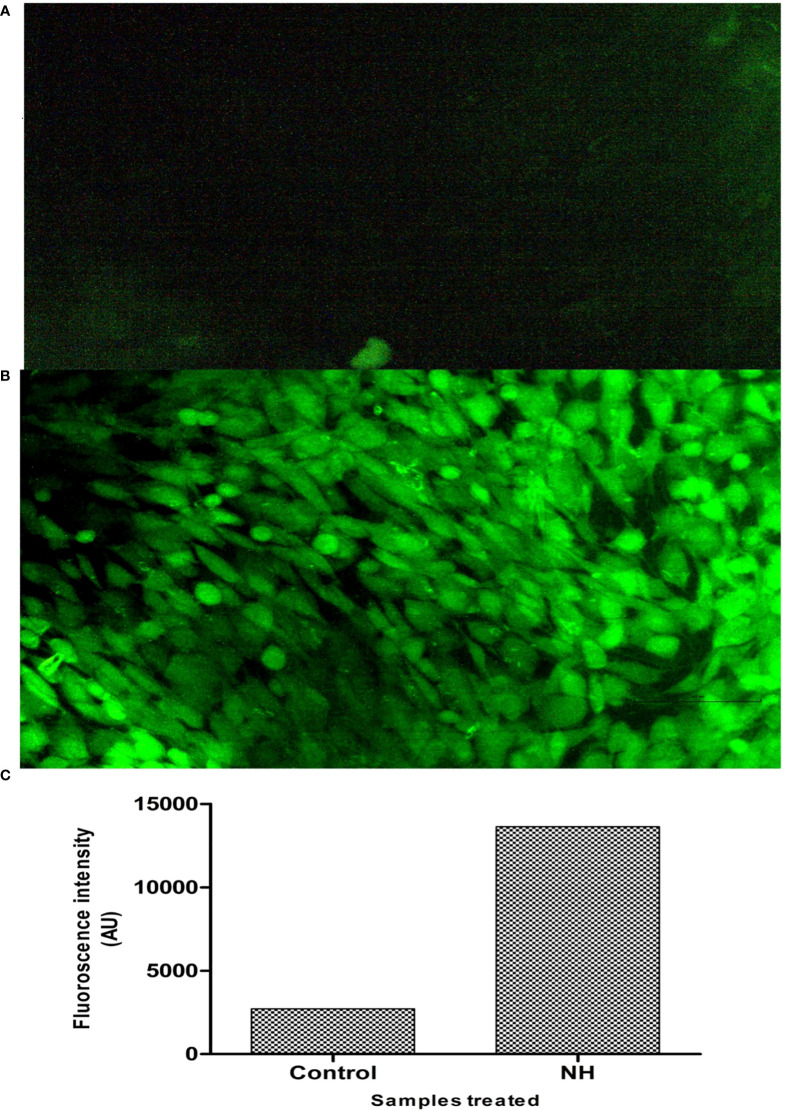
Deduction of cellular ROS by DCFDA staining assay. **(A)** Fluorescence image of control MG63 cells. **(B)** Fluorescence image of hexane extract treated MG63 cells. **(C)** Spectroscopic fluorescence intensity measurement of n-hexane–treated MG63 cells compared with control.

LDH assay is a sensitive and reliable assay to detect the extent of cell damage in the treated cells indicated by the release of lactate dehydrogenase enzyme into the medium. These enzymes catalyze pyruvate conversion to lactate in the presence of NADH (40). The cell membrane damage of MG63 cells was assessed by the method of DGKC upon the treatment of NH extract. After incubation for 24 h, the culture supernatant of 50 µL was collected and mixed with 1 mL of working reagent, and OD was recorded at 340nm. The result is presented in **Figure 4**. The result denotes that the extent of cell damage was increased in response to dose concentrations. At higher concentrations, the cell damage was high, indirectly reflecting the leakage of the high level of LDH into the medium. This indicates the phenomenon of cell death by apoptosis induced by NH extract.

#### 3.2.4 *In vitro* ROS measurement using DCFDA staining

**Figure 5:**
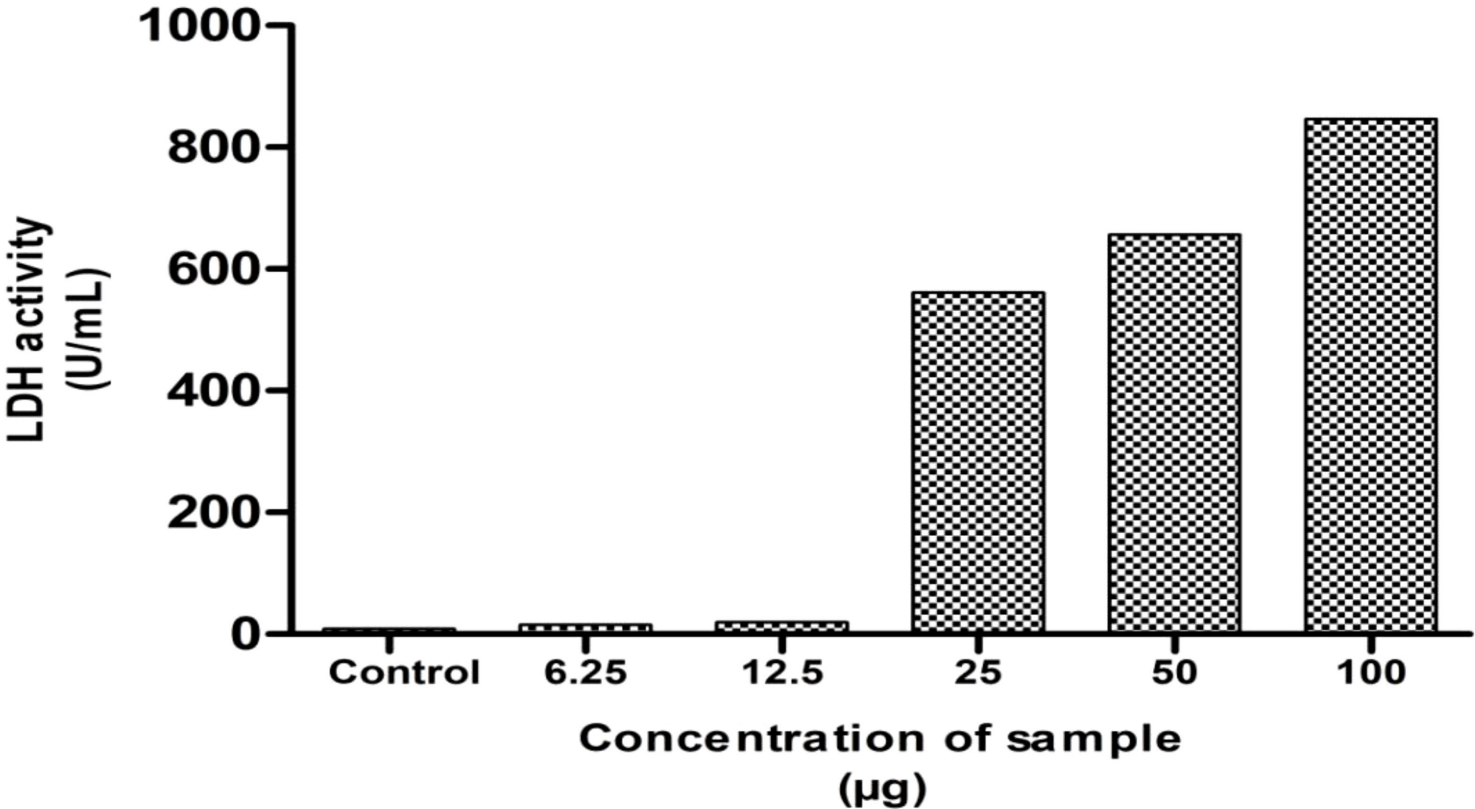
Lactate dehydrogenase assay (LDH) of the hexane extract against the MG63 cell line. The results showed the significant cytotoxicity at various concentrations.

Excessive cellular-level production of ROS damages the cell molecules (DNA/RNA/protein), eventually leading to cell death (41). To confirm that NH extract induces apoptosis in the MG63 cells by the ROS production upon the treatment, we investigated the intracellular production of ROS in the MG63 cells by the DCFDA staining method. Cellular esterases deacetylated the DCFDA to a non-fluorescent compound, which ROS later oxidizes into 2′,7′-dichlorofluorescein (DCF), emitting green fluorescence (42). Carboxy-H_2_DCFDA is non-fluorescent, but, in the presence of ROS, it becomes green fluorescent when this reagent is oxidized. The result is presented in **Figure 5**. The images denote that NH extracts induce the production of ROS by which the cells undergo the apoptosis process. The intensity of ROS production in control and treated cells was expressed in AU: control, 2,722.13; and treated cells, 13,363.12. It is thereby certain that NH extract induces apoptosis by the intracellular production of ROS in the MG63 cells.

### 3.3 Cell cycle analysis

**Figure 6:**
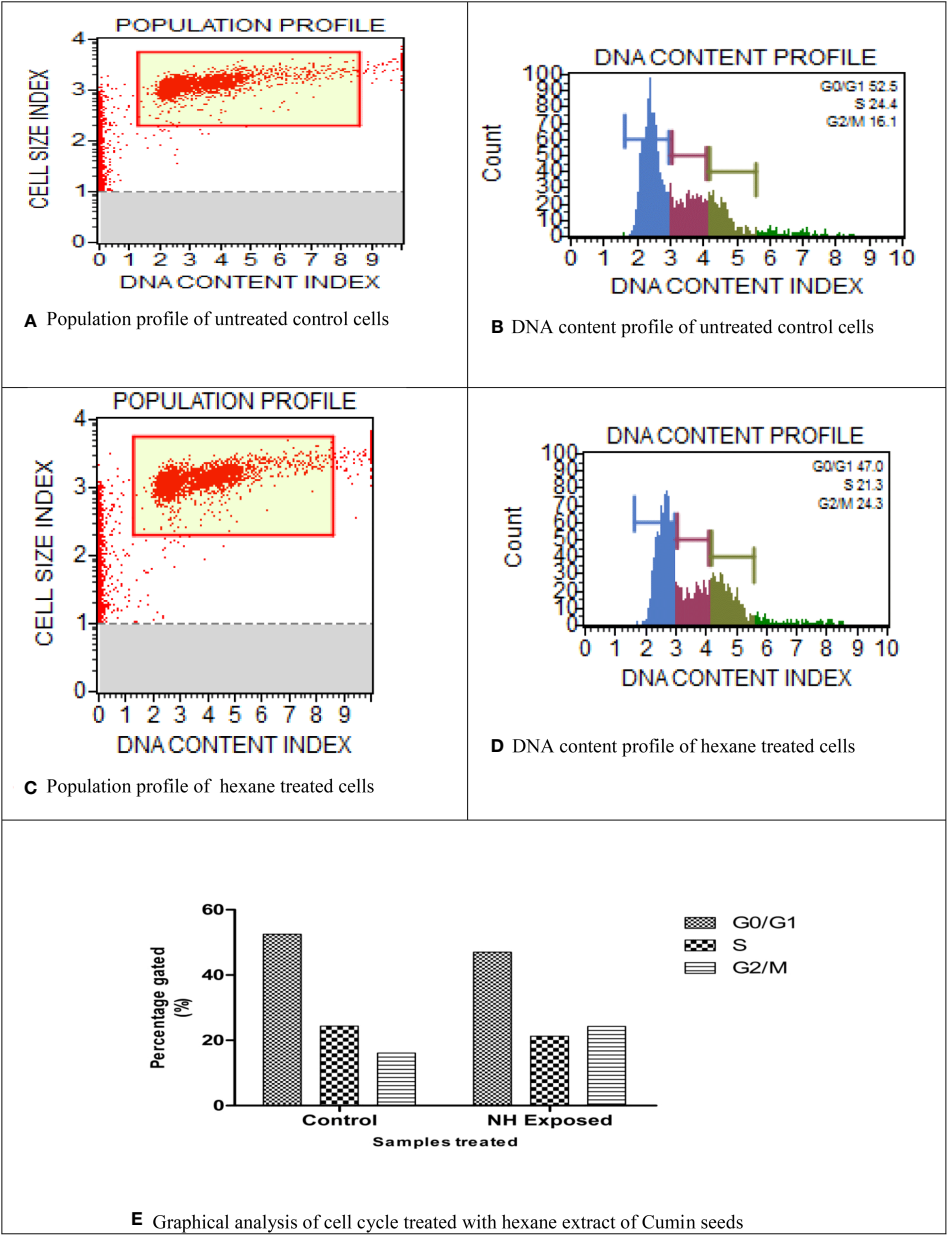
Cell cycle analysis of n-hexane–treated MG63 cells. **(A)** Population profile of untreated control cells. **(B)** DNA content profile of untreated control cells. **(C)** Population profile of hexane-treated cells. **(D)** DNA content profile of hexane-treated cells. **(E)** Graphical analysis of cell cycle treated with hexane extract of cumin seeds.

Using flow cytometry analysis, the inhibitory effect of NH extract in the MG63 cell cycle phases was investigated. **Figures 6A–E** show a definite change in the cellular DNA of MG63 cells. The G0/G1 phase (52.5%) showed a significant buildup of the cell population in the control cells, followed by the S phase (24%). By contrast, the cell population of the treated cells decreased significantly in the G0/G1 phase (47%) and the S phase (21%). In addition, we saw a significant alteration in the G2 cell cycle. To explain the above observation, we infer that the NH extract residue underwent apoptosis in the MG63 cells. By inducing apoptosis and cell cycle arrest, respectively, plant extract kills cells. Separating apoptotic cells with fragmented DNA from those that have lost cellular DNA can be done using flow cytometry (43).

### 3.4 Scratch wound healing assay

**Figure 7:**
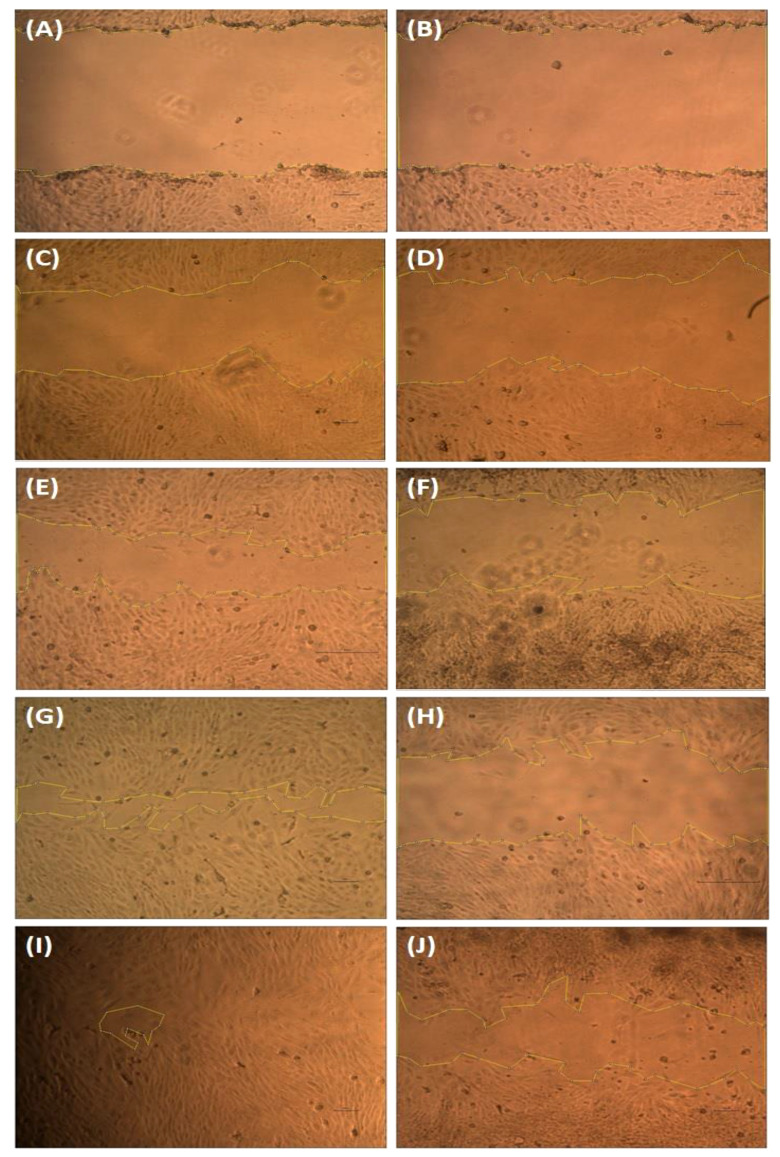
Wound healing assay. The MG63 cells were scratched and treated with hexane extract. After 24 h, the images were captured using an Olympus CKX41 microscope with ×4 magnification. Control images **(A, C, E, G, I)** and treatment cells **(B, D, F, H, J)**.

**Figure 8:**
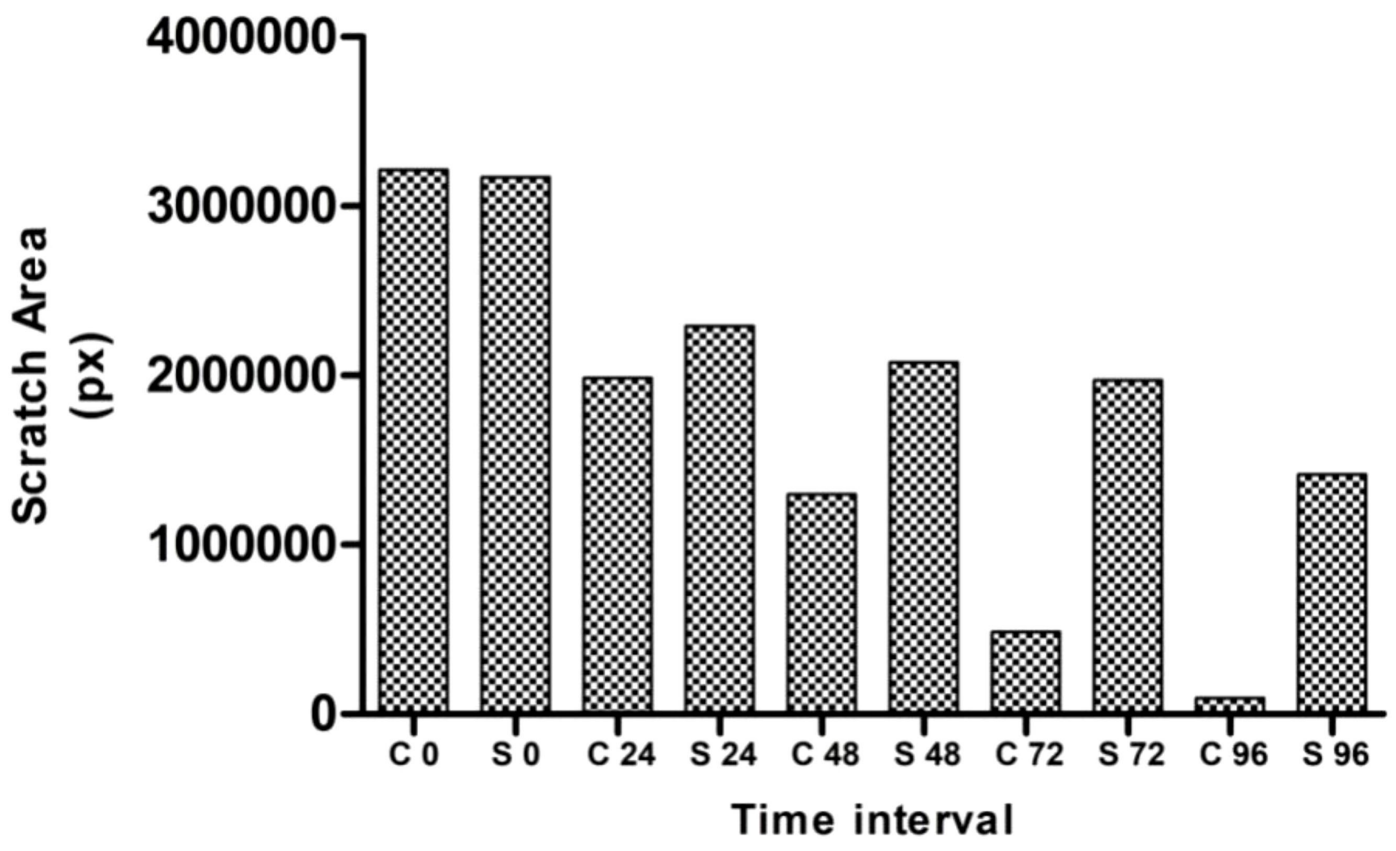
Graphical representation depicting the migration assay (C, control; S, hexane extract).

According to Krakmal et al. (44), invasion and migration of cancer are critical stages in the malignancy of cancer. In this work, we evaluated the MG63 cells’ ability to repair wounds *in vitro*. The activity outcomes are shown in **Figure 7**. As anticipated, the hexane extract successfully and slowly prevented MG63 cells from migrating. The control cells moved more quickly than the hexane-treated cells in comparison to the control. The influence of wound closure rate at various time intervals is shown by the graph plotted from the image (**Figure 8**). On the basis of the investigation, it can be concluded that hexane extract causes the MG63 to become immobile, disturb cells and, ultimately, induces apoptosis.

### 3.5 Clonogenic assay

**Figure 9:**
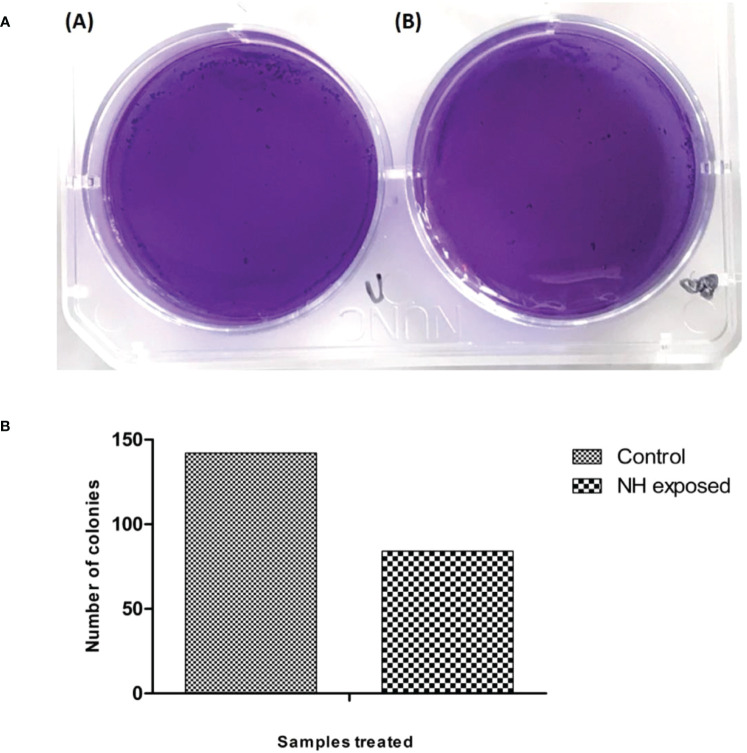
**(A)** Clonogenic assay of MG63 cells treated with n-hexane extract: (i) control; (ii) treated cells. **(B)** Graphical representation of clonogenic assay in hexane-treated MG63 cells.

Another important parameter to assess the therapeutic efficacy of the drug is the clonogenic assay. This assay determines the drug’s ability to retain the cell from forming colonies, thus reducing the survival of the cancer cell line. The present study employed hexane extract at a concentration of 86 µg/mL for the clonogenic assay. The assay result is presented in **Figure 9**; the image conferred that the extract after the incubation reduced the colonies’ growth significantly. These results indicate that the hexane extract of cumin seed has antiproliferative activity against bone cancer.

### 3.6 Identification of phytoconstituents

**Figure 10:**
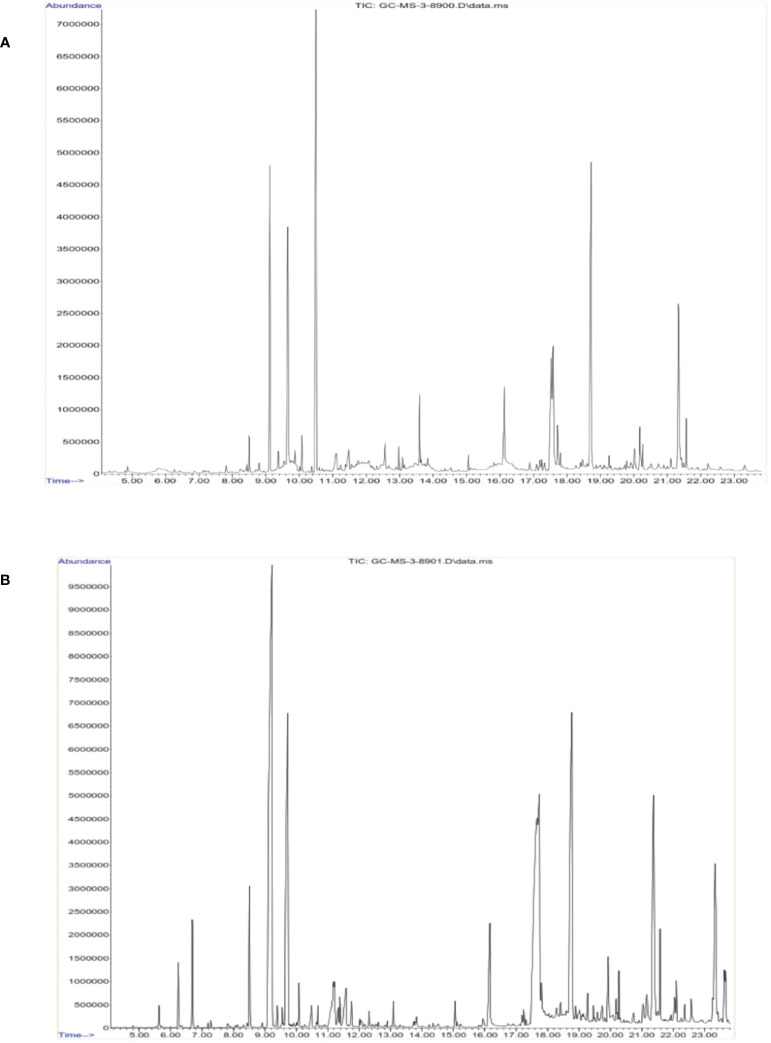
Identification of functional chemical constituents present in cumin seed extract using GC-MS. **(A)** Methanol extract. **(B)** n-Hexane extract.

On the basis of the above findings from the antibacterial and anticancer activities, it is deemed that methanolic and hexane extracts performed superior activities in bacterial and cancer cells. Hence, we qualitatively identified the chemical constituents present in the extract (**Figure 10**). The identified molecules were compared with the NIST database and tabulated the compounds (**Tables 2A**, **B** ). From the GC-MS analysis, the M extract contains abundantly phthalic acid (21%), whereas, in hexane extract, the major compound is propanal, 2-methyl-3-phenyl- (23%). Phthalic acid is reportedly present in various plants and possesses strong antibacterial properties (45). 2-Methyl-3-phenyl- is a derivative of methyl esters reportedly present in plants with various biological properties (46). The GC-MS analysis postulates that M and hexane extract contains various bioactive molecules that claim to be antimicrobial, anticancer, arthritis, immunosuppressants, and others (47–52). Thus, from the analysis, it is inferred that the chemical constituents actively synergistically spur the bactericidal and anticancer effects (53). However, the isolation of important abundance compounds in the extract must be studied to investigate the cancerous activity.

**Table 2A:** Qualitative GC-MS analysis of methanol extract.

Peak number	Name of the compound	Time (in min)	Percentage (%)	Abundance
1	d-Galactitol	5.787	1.28	16
2	Pulegone	8.497	1.09	53
3	Propanal, 2-methyl-3-phenyl-	9.120	10.59	98
4	Cyclohexene, 3-methyl-6-(1-methylethylidene)-	9.375	0.65	91
5	2-Methoxy-4-vinylphenol	9.653	8.45	76
6	2-Methoxy-4-vinylphenol	9.864	1.11	93
7	1,4-Cyclohexadiene-1-methanol, 4-(1-methylethyl)-	10.075	1.01	72
8	Phthalic acid	10.497	21.73	50
9	Benzoic acid, 4-(1-methylethyl)-	11.108	1.37	93
10	Hepta-2,6-dienoic acid	11.486	1.13	56
11	.beta.-D-Glucopyranose	12.075	0.44	53
12	1,4-Anhydro-d-galactitol	12.441	0.48	64
13	3-Methylsalicylhydrazide	12.564	1.09	76
14	Methanol	12.975	0.55	50
15	Ethanone, 1,1′,1″-(1,3,5-benzene…	13.597	1.90	49
16	4-Isopropylcinnamic acid	13.841	0.63	98
17	Cyclooctane	15.052	0.43	91
18	n-Hexadecanoic acid	16.130	3.28	87
19	9,12-Octadecadienoic acid (Z,Z)	17.530	6.68	99
20	6-Octadecenoic acid	17.585	5.33	99
21	Octadecanoic acid	17.719	1.39	99
22	1H-Indene, 2,3,3a,4,7,7a-hexahyd.	18.718	15.23	35
23	4-Amino-5-imidazolecarboxamide hydrochloride	20.018	1.03	38
24	Hexadecanoic acid 2-hydroxy-1-(hydroxymethyl) ethyl ester	20.174	1.57	62
25	Propanedinitrile, dicyclohexyl-	20.263	0.62	59
26	Cyclohexene, 5-methyl-3-(1-methylethenyl)-	21.096	0.47	46
27	Z,E-7,11-Hexadecadien-1-yl acetate	21.329	8.20	83
28	3-Methylbut-2-enoic acid	21.562	1.41	35
29	trans-2-Dodecen-1-ol, pentafluoropropionate	22.218	0.44	56
30	1H-Benzimidazol-2-amine	23.318	0.44	46

**Table 2B:** Qualitative GC-MS analysis of n-hexane extract. The compounds were identified using NIST library.

Peak number	Name of the compound	Time (in min)	Percentage (%)	Abundance
1	.beta.-Pinene	5.631	0.38	97
2	Benzene, 1-methyl-3-(1-methylethyl)-	6.242	0.94	95
3	1,4-Cyclohexadiene	6.686	1.57	94
4	1,3,3-Trimethylcyclohex-1-ene-4-carboxaldehyde, (+,-)-	8.508	2.11	60
5	Propanal, 2-methyl-3-phenyl-	9.231	23.80	97
6	p-Menth-2-en-7-ol	9.397	0.36	87
7	1-Cyclohexene-1-carboxaldehyde	9.553	0.32	94
8	1,5,6,7-Tetrahydro-4-indolone	9.719	10.77	62
9	1,4-Cyclohexadiene-1-methanol, 4-(1-methylethyl)-	10.086	0.60	49
10	3,3-Dimethyl-6-methylenecyclohexene	10.486	0.48	60
11	Benzoic acid, 4-(1-methylethyl)-	11.186	2.65	94
12	Cycloprop-2-enecarbonic acid	11.341	0.45	38
13	1,6,10-Dodecatriene, 7,11-dimethyl-3-methylene-	11.386	0.35	96
14	2,4-Cyclohexadiene-1-carboxylic acid	11.586	1.41	35
15	Tricyclo[5.4.0.0(2,8)]undec-9-ene, 2,6,6,9-tetramethyl-, (1R,2S,7R,8R)-	11.764	0.47	52
16	Carotol	13.097	0.35	91
17	4-Isopropylcinnamic acid	13.830	0.26	98
18	Pyrene, hexadecahydro-	15.052	0.35	46
19	n-Hexadecanoic acid	16.163	2.63	99
20	9,12-Octadecadienoic acid (Z,Z)-	17.663	14.57	99
21	cis-Vaccenic acid	17.807	0.56	97
22	Ethyl 2-(O-nitrophenylhydrazono).	18.774	11.30	41
23	2-Cyclohexen-1-ol, 2-methyl-5-(1-methylethenyl)-, cis-	19.018	0.35	55
24	Benzene, 2-methoxy-4-methyl-1-(1-methylethyl)-	19.129	0.31	43
25	Phthalic acid, hexyl tridec-2-yn-1-yl ester	19.274	0.49	35
26	2-Pentenoic acid, 2-methyl-, (E)	19.740	0.40	64
27	9,17-Octadecadienal, (Z)-	19.929	1.40	76
28	Hexadecanoic acid 2-hydroxy-1-(hydroxymethyl) ethyl ester	20.185	0.43	86
29	3-Methylbut-2-enoic acid	20.274	0.65	42
30	2-Methyl-4,5-diphenyl-4,5-dihydro	21.029	0.55	38
31	Stigmasterol	21.151	0.86	94
32	2,3-Dihydroxypropyl elaidate	21.373	7.32	90
33	6-Octadecenoic acid	21.496	0.28	49
34	3-Methylbut-2-enoic acid, 3,5-di.	21.585	1.22	35
35	.gamma.-Sitosterol	22.040	0.59	97
36	Hydrazine	22.096	0.54	49
37	Benzene, octyl-	22.362	0.28	47
38	Hexadecane, 1-iodo-	22.573	0.47	96
39	Benzene, 1,1′-(1,1,2,2-tetramethyl-1,2-ethanediyl)bis[4-methyl-	23.329	5.46	53
40	Benzene, (2-methylpropyl)-	23.629	1.75	53

The compounds were identified using NIST library.

## 4 Conclusion

The present study manifests the therapeutic properties of cumin seed extract against MDR strains and bone cancer cells. Upon the solvent extraction of the cumin seed, the extracted residue was assayed against the MDR pathogen *B. flexus*, *B. filamentosus*, *P. stutzeri*, and *A. baumannii*. As a result, the methanolic extract performed better bactericidal activity against the MDR strains. Furthermore, we substantially evaluated cumin seed extract’s anticancer and antiproliferative activities in MG63 cell line. The MTT assay was investigated in MG63 cell line. Among the solvent extracts, hexane triggers stupendous cytotoxic properties in MG63 cells. Moreover, hexane extract was sequentially assessed in AO/EB staining; the findings portrayed that hexane extract induced apoptosis in the MG63 cells. The LDH assay and DCFDA staining results also confirm the induction of apoptosis in the MG63 cells. The hexane extract arrests the S phase of the cell cycle revealed by flow cytometry analysis. In addition, we have studied the scratch wound healing assay in the MG63 cells. The hexane extract prevents the migration of the cells when compared with the control. The hexane extracts also inhibit the formation of colonies assessed by clonogenic assay. On the basis of the above results and interpretation, we have qualitatively determined the chemical constituents present in the methanolic and hexane extract using GC-MS. The analysis reveals the chemical constituents that proclaim to have various therapeutic properties. Thus, we can conclude that cumin seed extract has stringent cancerous activity in the MG63 cells and can be formulated as a chemotherapeutic agent in the near future.

## Data availability statement

The original contributions presented in the study are included in the article/supplementary material. Further inquiries can be directed to the corresponding authors.

## Author contributions

RC: Conceptualization, Writing – original draft. KM: Resources, Writing – review & editing. SC: Investigation, Writing – review & editing. OG: Methodology, Writing – review & editing. SH: Methodology, Writing – review & editing. JJ: Methodology, Writing – review & editing. EG: Methodology, Writing – review & editing. NA: Validation, Writing – review & editing. AA: Supervision, Writing – review & editing. SP: Supervision, Writing – review & editing. HJ: Conceptualization, Writing – original draft.
